# Schizophrenia long-acting antipsychotics initiation index (SLAAII)

**DOI:** 10.1192/j.eurpsy.2021.2149

**Published:** 2021-08-13

**Authors:** P. Ifteni, A. Teodorescu

**Affiliations:** Faculty Of Medicine, Transilvania University of Brasov, Brasov, Romania

**Keywords:** schizophrenia, long-acting antipsychotics, initiation

## Abstract

**Introduction:**

Background In individuals with schizophrenia, long-acting injectable antipsychotics (LAIs) have been shown to be beneficial in preventing relapse. An important issue in these individuals is poor medication adherence, which can negatively affect outcomes. Although currently underutilized in comparison with oral antipsychotics, LAIs can be an important treatment option for addressing the high rates of poor adherence to medication in individuals with schizophrenia. There is a lack of published evidence and treatment guidelines on optimal strategies for the initiation of treatment with LAIs, which would at least partly explain why LAIs remain underutilized.

**Objectives:**

Aims The aim of this report is to present an index for initiation of LAI in schizophrenia.

**Methods:**

A restrospective chart review of a cohort of 1000 consecutive patients hospitalized with schizophrenia in Clinical Hospital of Psychiatry and Neurology Brasov, Romania, between 2011 and 2019. The number and reasons of LAIs initiation were evaluated.

**Results:**

Rezults The results shows a reduced number of LAIs initiation and led to the realization of an index entitled Schizophrenia long-acting antipsychotics initiation index (SLAAII) with 6 domains (age, duration of illness, number of relapses, response to oral treatment and antipsychotic available formulation), each with 3 response variants rated with 5 points, 3 points and 1 point. The maximum posible score is 30 points and minimum 6 points. A score above 20 points is a strong indication for LAI initiation.

**Conclusions:**

Schizophrenia long-acting antipsychotics initiation index (SLAAII) could be a very useful tool to facilitate the initiation of LAI treatment in patients with schizophrenia.

**Disclosure:**

No significant relationships.

